# Thapsigargin—From Traditional Medicine to Anticancer Drug

**DOI:** 10.3390/ijms22010004

**Published:** 2020-12-22

**Authors:** Agata Jaskulska, Anna Ewa Janecka, Katarzyna Gach-Janczak

**Affiliations:** 1 Institute of Organic Chemistry, Lodz University of Technology, Żeromskiego 116, 90-924 Lodz, Poland; agata.jaskulska@dokt.p.lodz.pl; 2 Department of Biomolecular Chemistry, Medical University of Lodz, Mazowiecka 6/8, 92-215 Lodz, Poland; anna.janecka@umed.lodz.pl

**Keywords:** thapsigargin, cytotoxin, anticancer activity, sarcoplasmic/endoplasmic reticulum Ca^2+^ ATPase, unfold protein response, apoptosis, prodrug, prostate-specific antigen, prostate-specific membrane antigen, mipsagargin

## Abstract

A sesquiterpene lactone, thapsigargin, is a phytochemical found in the roots and fruits of Mediterranean plants from *Thapsia* L. species that have been used for centuries in folk medicine to treat rheumatic pain, lung diseases, and female infertility. More recently thapsigargin was found to be a potent cytotoxin that induces apoptosis by inhibiting the sarcoplasmic/endoplasmic reticulum Ca^2+^ ATPase (SERCA) pump, which is necessary for cellular viability. This biological activity encouraged studies on the use of thapsigargin as a novel antineoplastic agent, which were, however, hampered due to high toxicity of this compound to normal cells. In this review, we summarized the recent knowledge on the biological activity and molecular mechanisms of thapsigargin action and advances in the synthesis of less-toxic thapsigargin derivatives that are being developed as novel anticancer drugs.

## 1. Introduction

Thapsigargin (Tg), a guaianolide-type sesquiterpene lactone, is abundant in the common Mediterranean weed *Thapsia garganica* (Apiaceae), known as “deadly carrot” due to its high toxicity to sheep and cattle. The skin-irritating properties, as well as the medical use of this plant, were known already in ancient times. The resin from the roots and fruits of *T. garganica* was used for centuries in folk medicine to treat several diseases, such as pulmonary diseases, female infertility, catarrh, fever, and rheumatism [1,2].

The skin irritating component, named Tg, was isolated from *T. garganica*, along with other structurally-related guaianolides, by Christensen and co-workers in 1978 [3,4], whereas the full structure and the absolute configuration of this compound was established in 1985 [5].

**Figure 1:**
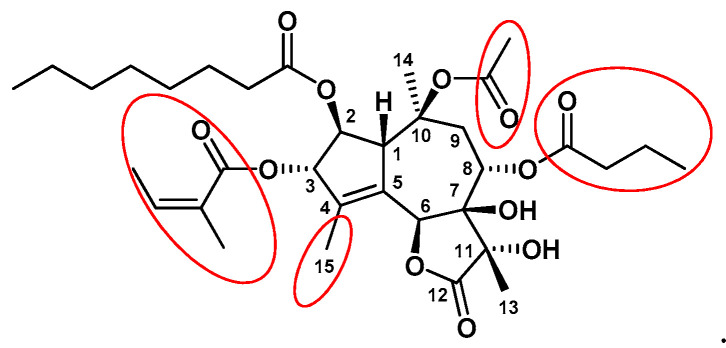
The chemical structure of Tg. Pharmacophoric groups important for biological activity are marked in red circles.

In terms of chemical structure, Tg consists of three fused rings, formed by annulation of a cycloheptane, cyclopentene, and a γ-lactone (Figure 1). The structural complexity of this carbon skeleton was challenging for organic chemists [2]. The first total synthesis of Tg from (*S*)-carvone, achieved in 42 steps with 0.6% overall yield, was reported by Ley’s group in 2007 [6,7]. Christensen and co-workers described a concise synthesis of Tg from nortribolide in 2015 [8]. Starting with (*S*)-dihydrocarvone Baran and co-workers performed a second, 11-step total synthesis in 2016 [9]. Most recently, in 2017, Evans’s group reported an efficient 12-step synthesis from commercially available (*R*)-carvone with 5.8% overall yield [10].

The interesting biological activities of Tg, especially high cytotoxicity, opened perspectives of its use as an anticancer agent, which caused a growing demand for this compound. The low-yield tedious isolation of Tg from *T. garganica* and laborious and expensive chemical synthesis made the availability of Tg very limited. The solution may lie in the development of production platforms for Tg, including agriculture of *T. garganica* plants and plant tissue cultures producing Tg, which are already in progress by several companies [1,11], as well as in new, cost-effective synthetic approaches [2].

Over the past 40 years, several excellent reviews describing Tg and its derivatives at different angles have been published [1,2,12,13,14]. In this review, we focused mostly on Tg’s biological activities and molecular mechanisms of action in cancer cells. We also discuss the development of novel Tg-based prodrugs which may become effective anticancer agents with limited side-effects.

## 2. Molecular Mechanism of Tg Action

### 2.1. Tg as a SERCA Pump Inhibitor

The sarco/endoplasmic reticulum Ca^2+^-ATPase (SERCA) pump, plays a crucial role in regulation of calcium homeostasis which is pivotal for cell signaling and cell survival [15,16]. SERCA pump is a P-type ATPase that uses the energy of ATP hydrolysis to pump Ca^2+^ ions from the cytoplasm into the sarcoplasmic reticulum (SR) lumen in skeletal and cardiac muscle cells, and into the endoplasmic reticulum (ER) of non-muscle cells, maintaining low cytosolic Ca^2+^ concentration [17]. Such compartmentalized distribution of Ca^2+^ in the cell is responsible for regulation of various cellular functions such as proliferation, contractility, protein folding, gene transcription, and apoptosis [15,18,19]. SERCA exists in several cell-type-dependent (tissue-specific) isoforms, encoded by three *ATP2A1-3* genes [20]. Despite the high percentage of their homology, they have different Ca^2+^ affinity and enzyme kinetics [17,21]. Impaired SERCA function leads to the elevation of intracellular Ca^2+^ levels and depletion of Ca^2+^ in ER stores, triggering ER stress and stress-induced apoptosis [20]. Dysregulation of Ca^2+^ metabolism may cause cardiovascular and muscular diseases, diabetes and also cancer [15,19,20].

On the other hand, SERCA inhibitors are proposed as novel therapeutic agents in cancer therapy [15,16]. The best known and most thoroughly studied representative of SERCA inhibitors is Tg [16,22]. Tg blocks SERCA ATPase activity at subnanomolar or low nanomolar concentrations [21,23] and at these concentrations has no influence on either the plasma membrane Ca^2+^-ATPase or Na^+^,K^+^-ATPase [24]. The SERCA pump transports two Ca^2+^ per hydrolyzed ATP molecule via an “alternating-access” mechanism, while the enzyme interconverts between two main conformational states E1 and E2 [25]. In E1 state, ATPase is activated by binding of two Ca^2+^ ions, in E2 state, two Ca^2+^ ions are released into the ER lumen. Therefore, E1 and E2 states are characterized by high and low affinity for Ca^2+^ ions, respectively. Tg interacts stoichiometrically with the SERCA pump in a calcium-free E2 conformational state, forming an irreversible, catalytically inactive ‘dead-end’ inhibitory complex, which prevents Ca^2+^ binding and ATPase activation [15,22,26].

### 2.2. Tg as an Endoplasmic Reticulum Stressor and Unfolded Protein Response (UPR) Inducer

The ER plays a key role in Ca^2+^ storage and dynamics and is a major site for protein synthesis, folding and maturation of eukaryotic cells [27]. Disturbances of ER functions, leading to protein folding defects and accumulation of unfolded or misfolded proteins in the lumen, results in ER stress [28]. To alleviate this stress, cells activate a network of signaling pathways known as the unfolded protein response (UPR) that, depending on the level of damage, restores protein homeostasis (adaptive UPR) or triggers apoptosis (apoptotic UPR) [28,29,30,31].

**Figure 2:**
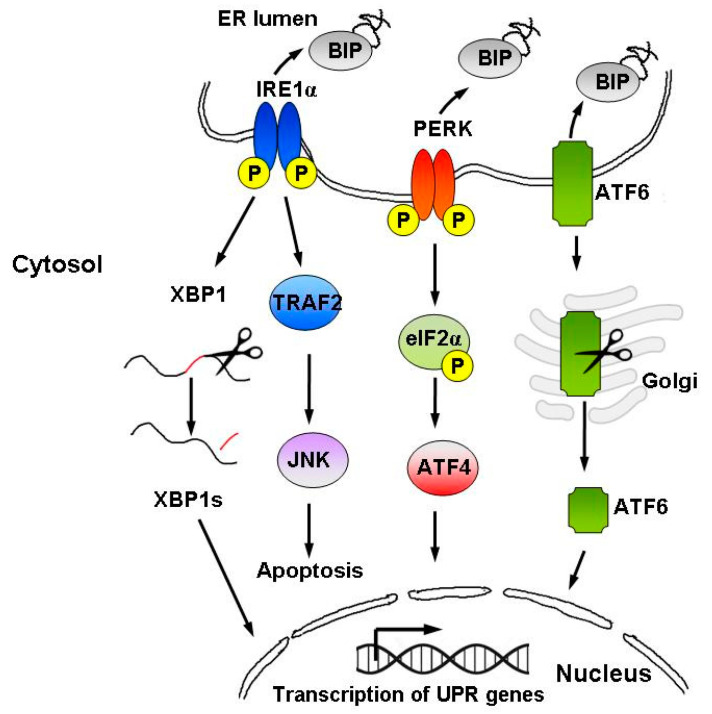
Schematic representation of the UPR signaling pathways. The UPR is activated by accumulation of unfolded proteins in the ER lumen, followed by BiP dissociation from the three ER stress sensors, i.e., IRE1α, PERK, and ATF6. The UPR instigates a transcriptional and translational response to ER stress in order to restore protein homeostasis. However, upon persistent ER stress, pro-apoptotic signaling is induced and the cell undergoes programmed cell death.

Signaling through the UPR increases protein folding, transport and ER-associated protein degradation (ERAD), while inhibiting protein synthesis [31]. The UPR comprises three parallel signaling branches controlled by transmembrane ER sensors: protein kinase R-like ER kinase (PERK), transcription factor-6 (ATF6) and inositol-requiring enzyme 1α (IRE1α) [27,32], as well as the downstream transcription factors: X-box-binding protein 1 (XBP1), activating transcription factor-4 (ATF4) and transcription factor C/EBP homologous protein (CHOP) [30] (Figure 2). In non-stressed cells, ER sensors remain in an inactive state due to their association with binding immunoglobulin protein (BiP, also known as GRP78). Upon ER stress and accumulation of unfolded or misfolded proteins, BiP dissociates from the three ER proteins, IRE1, PERK, and ATF6, activating their respective UPR pathways in order to restore homeostasis [27,32].

However, when ER stress is persistent and unresolved, UPR triggers specific apoptotic pathways to eliminate severely damaged cells [33,34,35]. ER stress-induced cell death may occur through activation of both intrinsic and extrinsic apoptotic pathways, involving Bcl-2 proteins (Bim, Noxa, and Puma) and tumor necrosis factor-related apoptosis-inducing ligand (TRAIL) receptors [30].

Chronic ER stress and defects in UPR signaling may lead to various diseases, including cancer, diabetes, neurodegenerative and autoimmune disorders [36,37]. UPR sits in the center of life-or-death decisions of the cell, activating either pro-survival or pro-apoptotic pathways. Hence, inducing cell death via ER stress and UPR might be a promising therapeutic strategy for killing cancer cells [38].

Tg is known as an ER stressor that initiates UPR-mediated apoptosis [35,39,40] but the contributions of various UPR components involved in cell death initiation remain unclear. In numerous studies on cancer cell lines (Table 1), it has been shown that death receptor 5 (designated DR5, or TRAIL-R2) and caspase-8 play an essential role in Tg-induced apoptosis via UPR (in a response to ER stress) [33,34,39,40,41,42].

Linder et al. demonstrated that in human prostate (LNCaP) and colorectal (HCT116) cancer cell lines exposed to Tg, microtubule-associated protein 1A/1B light chain 3B (LC3B), normally associated with autophagy, was required for optimal activation of caspase-8 [39]. The authors showed that PERK, and its downstream transcription factors: ATF4 and CHOP, were necessary for Tg-induced cell death but surprisingly they acted in parallel rather than as a linear pathway. The expression of both DR5 and LC3B was controlled by ATF4 and CHOP, whereas PERK was required for cell apoptosis but acted via other pathways [39].

The involvement of CHOP in up-regulation of DR5 and promotion of apoptosis in Tg-treated cells was observed also by other authors [33,41,43]. Chen et al. demonstrated that up-regulation of DR5 in Tg-treated melanoma cells was cooperatively mediated by IRE1α and ATF-6-signaling pathways, along with the transcription factor CHOP [43]. Moreover, they indicated that Tg might be useful in sensitizing melanoma cells to TRAIL-induced apoptosis [43]. Similarly, Ma and colleagues showed that Tg sensitized esophageal squamous cell carcinoma cell line (ESCC) to TRAIL-induced apoptosis via the TRAIL-DR5-AMP activated protein kinase (AMPK) pathway [41]. Detailed studies revealed that Tg-induced ER stress increased CHOP expression, thus up-regulating DR5. TRAIL/DR5 activation induced apoptosis in ESCC cells, which was mediated by oxidative stress via AMPK phosphorylation [41]. Additionally, inducing ER stress, Tg could also directly activate AMPK phosphorylation, which further promoted apoptosis [41]. TRAIL is a member of tumor necrosis factor (TNF) family which, when bound to DR5, can cause apoptosis in a variety of cancer cell types, while sparing normal cells [47]. Therefore, using Tg as an ER stress inducer may be beneficial for improving the efficacy of TRAIL-based anti-cancer agents [41,43].

IRE1α is yet another UPR sensor, whose role in response to ER-stress is complex and not fully elucidated. It controls both cell survival and the decision to execute apoptosis. Activation of IRE1α leads to production of the alternatively spliced isoform of XBP1 (XBP1s), contributing to cell survival during mild, resolvable ER-stress, whereas activation of c-Jun N-terminal kinase (JNK) late in the ER stress response results in cell death [27,48].

The involvement of JNK signaling pathway in Tg-mediated ER stress apoptosis was confirmed in several cancer cell lines and in the in vivo models [39,44,48]. Wu et al. showed that promotion of apoptosis in adrenocortical carcinoma (ACC) cells and suppression of ACC xenograft growth in mice treated with Tg was caused by up-regulation of JNK signaling-related markers [44]. Tg significantly enhanced expression of JNK signaling-related genes (JNK, ATF6, PERK, LC3B, and Bcl-2) and proteins (JNK, MAPK, ERK) and their phosphorylated forms, suggesting activation of JNK/MAPK/ERK signaling pathway in Tg-induced apoptosis in ACC [44]. Recently, it has been suggested that two functionally distinct phases of JNK signaling—An early pro-survival and late pro-apoptotic phase exist in ER stress response and Tg was shown to enhance JNK activity in these both phases [39,48]. Brown et al. [48] demonstrated that the initial phase of JNK activation in response to Tg-induced ER stress depended on both IRE1α and tumor necrosis factor receptor-associated factor 2 (TRAF2), and produced anti-apoptotic signals, protecting cells from executing apoptosis. On the other hand, Linder et al. [39] showed that IRE1-XBP1 was required for the second-phase activation of JNK by Tg, which promoted caspase activation and apoptosis in a cell type-dependent manner [39]. The authors found that Tg induced apoptosis in prostate cancer LNCaP cells, sensitive to pro-death effects of JNK, but not in JNK independent colon cancer HCT116 cells [39].

Recently, Chidawanyika et al. [45] investigated genes engaged in Tg-induced apoptosis. Using a near-haploid cell line HAP1, they identified a novel gene, SEC24A, essential for Tg-induced cytotoxicity in HAP1 cells. They showed that the ability of SEC24A to facilitate ER stress-induced cell death is specific to Tg and that SEC24A is a crucial mediator of Tg-induced UPR and apoptosis.

### 2.3. Correlation Between ER Ca^2+^ Depletion, UPR and Cell Death

As a result of blocking the SERCA pump, Tg depletes ER Ca^2+^ stores and activates UPR, which may eventually result in cell death [34,35,39]. However, the exact mechanism by which SERCA inhibition induces UPR and apoptosis is not understood. Seghal et al. [35] investigated the correlation between the ability of Tg to deplete ER Ca^2+^ stores and their detrimental effects on cell viability. The authors showed that Tg strongly inhibited SERCA1a-mediated Ca^2+^ transport. They also found that, in prostate (PC3, and LNCaP) and breast cancer (MCF-7) cells, one-day exposure to Tg caused extensive drainage of the ER Ca^2+^ stores. This Ca^2+^ depletion was followed by markedly reduced cell proliferation rates and morphological changes that developed over 2–4 days and culminated in cell death. The further studies in PC3 cells focused on extent and duration of ER Ca^2+^ depletion induced by Tg required for a apoptosis-inducing UPR [46]. The gradual decrease in the total ER Ca^2+^ levels observed with Tg (1-3 nM) correlated with decreased PC3 cell proliferation in the absence of UPR activation and without succumbing to cell death. The authors showed that a partial depletion of ER Ca^2+^ is sufficient to reduce cell proliferation. However, cells could tolerate strong and sustained decreases in ER Ca^2+^ without showing any signs of UPR. At higher cytotoxic concentrations of Tg (> 3 nM), the authors observed a gradual, caspase-dependent increase in cell death, which was likely to be caused by UPR, since an enhanced levels of XBP1s, ATF4, CHOP, BiP, and LC3B were observed. Szalai et al. [46] concluded that UPR activation and cell death required an extreme overall ER Ca^2+^ depletion, obtained at substantially higher Tg concentrations than those needed to strongly decrease ER Ca^2+^ levels.

Some authors indicated that increases in cytosolic Ca^2+^ levels, mediated via store-operated Ca^2+^ entry (SOCE), were required for ER Ca^2+^ depletion-induced cell death [49,50]. However, more recently it has been shown that apoptosis induced by Tg was not accompanied by the increase in cytosolic Ca^2+^ levels, as implied previously. The knock-down of two key SOCE components, calcium release-activated calcium channel protein 1 (Orai1) and sensor stromal interaction molecule 1 (STIM1), did not reduce Tg cytotoxicity in PC3, LNCaP or MCF-7 cells, indicating that SOCE-mediated increase in cytosolic Ca^2+^ was not critical for Tg-induced cell death [35]. Moreover, Tg produced maximal cell death at concentrations that were substantially lower than those needed to induce detectable increases in cytosolic Ca^2+^ levels [35,51].

Summing up, it has been shown that Tg-induced apoptosis is initiated by ER Ca^2+^ depletion and a sustained UPR activation, whereas high cytostolic Ca^2+^ concentration and SOCE are not required to start the cell death events [35,46].

## 3. Structure-Activity-Relationship Studies of Tg Analogs

**Figure 3:**
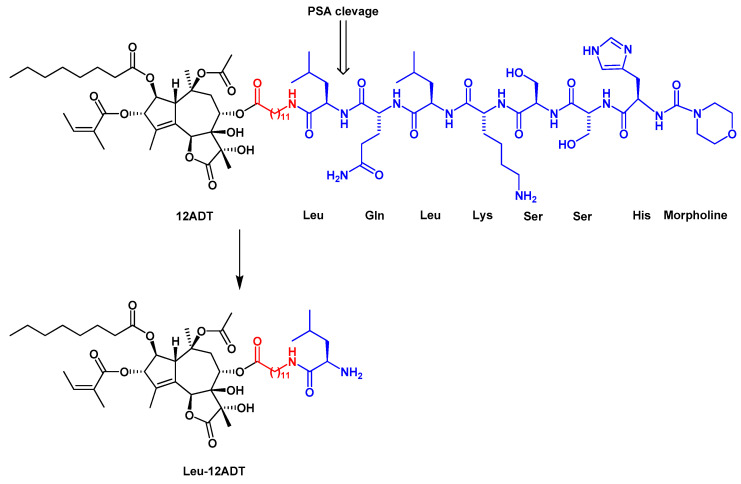
The structure of the G115 prodrug and the product of its PSA cleavage, Leu-12ADT. The linker is marked in red.

**Figure 4:**
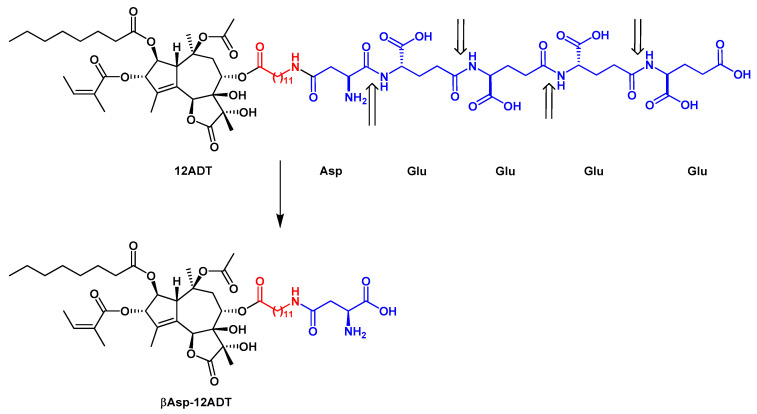
The structure of G202 prodrug and the major product of its PSMA cleavage, βAsp-12ADT. The linker is marked in red. The PSMA cleavage sites are indicated by arrows.

Detailed structure-activity relationship studies were performed to establish the effect of each functional group of Tg on its activity as a SERCA inhibitor [13]. According to the obtained pharmacophoric model, three acyloxy groups (at C-3, C-8, C-10), the methyl group at C-4 and the lactone ring interact with the backbone of SERCA pump via hydrophobic interactions [52,53,54] (Figure 1). The hydrophobic chain of the octanoyloxy group at C-2 extends into the lipid phase of a cell membrane forming a weak interaction with ATPase [54]. Detrimental for Tg activity was the inversion of the configuration at C-3 and C-8 [13,52], while long, flexible chains of acyloxy groups at C-8 did not reduce Tg’s inhibitory potency against SERCA. Therefore, modifications at C-8 became of particular interest in the design of new analogs. Replacing the butanoiloxy group at C-8 by a 12-aminododecanoiloxy moiety gave 8-(12-aminododecanoyloxy)thapsigargin, which was further modified by the attachment of amino acid residues to form Leu-12ADT (Figure 3) and βAsp-12ADT (Figure 4) [35,51,55,56].

## 4. Development of Tg-Based Prodrugs

Through the inhibition of SERCA pump, Tg induces cancer cell apoptosis in a proliferation-independent manner [55,57]. Most of the commonly used chemotherapeutics, such as paclitaxel, doxorubicin, or 5-fuorouracil, kill only rapidly-proliferating cells and, as a consequence, are ineffective against cancers that have a low rate of proliferation (prostate cancer). Tg has the ability to cause apoptosis in both proliferative and quiescent phases of the cell cycle [57]. However, the presence of SERCA pump in almost all kind of cells and its essential role for cell survival, makes Tg a general cell toxin [55,57]. This high toxicity excludes Tg as a drug candidate. To overcome this limitation and direct the cytotoxicity of Tg towards cancer cells, sparing normal tissue, the Tg-based pro-drug strategy has been developed [1,14,55,56,57,58].

Prodrugs are inactive compounds that, in the body, are transformed into pharmacologically active therapeutics [59]. The Tg-based prodrug strategy utilizes the proteolytic enzymes that are preferentially expressed on the surface of cancer cells or are secreted by cells of solid tumors. The conjugation of Tg with substrates for such enzymes masks cytotoxic activity of Tg, producing prodrugs that can be cleaved by a tissue-specific proteases expressed only in cancer cells [55,56,57,58].

Prostate-specific antigen (PSA) is a serine protease, produced and secreted only by the prostate luminal epithelial cells in both normal and malignant prostate tissue [55,60]. Extracellular fluid surrounding prostate cancer contains a high level of enzymatically active PSA, which is inactivated in the blood serum [61]. Prostate specific membrane antigen (PSMA) is a carboxypeptidase, which is overexpressed in prostate cancer and in most of endothelial cells in the vasculature of various tumor types, but not in normal vascular endothelium [56].

By coupling Tg derivatives to peptide carriers, which are substrates either for PSA or PSMA, protease-activated prodrugs, selectively affecting prostate cancer cells, were created [55,56,57]. The successful examples of such approach are two prodrugs, G115 [55,57], designed for the treatment of prostate cancer, and G202, selectively targeting the neovascular tissue in blood vessels of various cancer types, including prostate, breast, and bladder cancers [56,62,63]. The difference between G115 and G202 is in the peptide sequences that were attached to Tg derivative, 12ADT (Leu-Gln-Leu-Lys-Ser-Ser-His-morpholine in G115 and Asp-Glu-Glu-Glu-Glu in G202). PSA cleavage of the peptide component of G115 resulted in the release of a lipophilic Leu-12ADT (Figure 3), selectively killing prostate cancer cells in vitro and in vivo, with minimal systemic toxicity to the tumor bearing animals [55,57]. In order to more effectively target prostate cancer cells, a cell-based Tg delivery platform with the use of mesenchymal stem cells (MSCs), displaying the cancer tropism, has recently been developed [64].

G202 was developed as a Tg-based PSMA-activated prodrug that potentially could be used to treat almost all types of solid tumors by targeting PSMA enzymatic activity within tumors and surrounding tumor vasculature [56]. Upon PSMA-mediated cleavage of the peptide carrier of G202, the cytotoxic analog βAsp-12ADT is released, which was shown to be ~60-fold more toxic in vitro to PSMA-positive cells compared to PSMA-negative cells (Figure 4).

In vivo experiments on a variety of human cancer xenografts in mice reveled that G202 significantly inhibited progression of prostate, breast and bladder cancers, while being minimally toxic for the host animals [56]. After compelling preclinical results (low toxicity in monkeys), G202 has entered clinical trials, under the name mipsagargin. Phase I clinical trials demonstrated that mipsagargin was well tolerated and had favorable pharmacokinetic profile in patients with advanced solid tumors, resistant to standard therapy [62]. Phase II clinical trials on mipsagargin are summarized in Table 2 (http://clinicaltrials.gov; November 2020).

**Figure 5:**
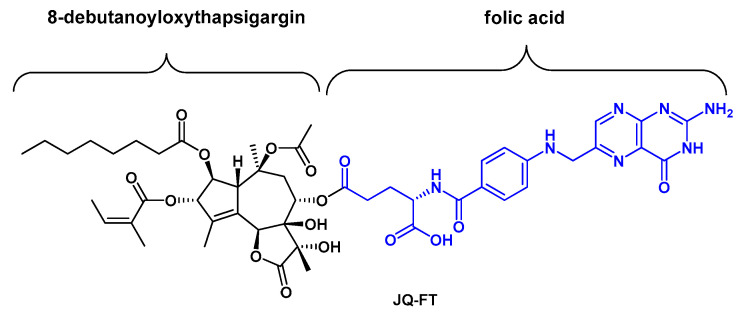
The structure of the Tg-folate prodrug, JQ-FT.

Antineoplastic activity of Tg-based prodrugs has been widely described in many types of solid tumors. However, there is almost no data on Tg application for leukemia treatment. Roti et al. demonstrated antileukemic activity of Tg in human T cell acute lymphoblastic leukemia (T-ALL) [65]. This is a high-risk leukemia known for frequent mutations in the *NOTCH1* gene [66]. NOTCH receptors (NOTCH1–4) function as ligand-activated transcription factors that regulate many aspects of normal cell development and tissue homeostasis. The authors showed that Tg-induced SERCA inhibition selectively impaired the maturation and activity of mutated NOTCH1 receptors in T-ALL cells, resulting in antileukemic activity of this molecule in vitro and in vivo [65]. Moreover, they noticed that low nanomolar concentration of Tg preferentially inhibited mutant NOTCH1 pathway in vivo, while wild-type NOTCH1 and NOTCH2 receptors were properly processed [65]. They concluded that mutated NOTCH1 might be more sensitive to reduced calcium availability than the wild-type NOTCH1, providing a therapeutic window for Tg. Although Tg had a promising anti-leukemic activity in mouse models of human T-ALL, its administration was limited due to its high toxicity [65]. To avoid nonspecific toxicity, further research was focused on the “targeted” delivery of Tg to T-ALL cells [67]. For this purpose the folate conjugation anticancer strategy [68], based on the high dependence of leukemia cells on folic acid metabolism and the presence of folate receptors (FRs) on their cellular surface, was used [67]. By conjugation of the folic acid to an alcohol derivative of Tg via a cleavable ester bond, Roti et al. [67] designed a Tg-folate prodrug JQ-FT (Figure 5). This molecule was recognized by FRs on the plasma membrane and delivered into leukemia cells. Moreover, using the mechanistic and translational models of T-ALL, NOTCH1 inhibition in vitro and in vivo was demonstrated. Folate-derived Tg delivery strategy is an interesting approach to enhance the therapeutic window of Tg, providing dual selectivity: leukemia over normal cells and NOTCH1 mutated over wild-type receptors [67]. However, further optimization and potency enhancement is required for clinical application of this molecule.

## 5. Conclusions and Future Perspectives

Natural products isolated from plants are an important source of chemotherapeutics, in particular, against cancer. However, in general, natural substances cannot be used directly as drugs, either because they have a narrow therapeutic window or because they possess undesired pharmacokinetic properties, such as poor absorption, low solubility, and/or fast metabolism. Therefore, optimization of the properties of natural phytochemicals is required. The solution lies in structural modifications of natural compounds or synthesis of their analogs with improved pharmacological properties.

Tg is a potent cytotoxin isolated from *T. garganica* over forty years ago. Tg induces apoptosis in a proliferation-independent manner by inhibiting the SERCA pump and emptying the ER Ca^2+^ stores. A barrier preventing the direct usage of Tg as an anticancer agent is its lack of selectivity, since Tg kills not only cancer but also normal cells. The unique properties of cancer cells were used to develop prodrugs that can transport Tg directly to cancer sites. G115 and G202 are examples of prodrugs obtained by conjugation of Tg to substrates of proteolytic enzymes, that are present only in cancer tissue. JQ-FT is an antileukemic prodrug developed by conjugation of folic acid and Tg derivative. Both described prodrug strategies represent an efficient approach to overcome Tg general cytotoxicity, and are in line with recent directions in targeted cancer therapy.

## Figures and Tables

**Table 1:** Cell death mode and key mediators involved in Tg action in various cancer cell lines.

Mode of Action/Key Proteins	Mediator	Cell Line	Ref
Apoptosis via DR5	CHOP	Colorectal carcinoma HCT116 Prostate cancer LNCaP, DU145 Ovarian cancer A2780	[33]
Apoptosis via DR5 and caspase 8		Colorectal carcinoma HCT116 Lung cancer SK-MES-1 Myeloma KSM11, RPMI-8226	[34]
Apoptosis		Prostate cancer PC3, LNCaP Breast cancer MCF-7	[35]
Apoptosis via DR5 and caspase-8	LC3B, CHOP, PERK, ATF4, IRE1	Prostate cancer LNCaP Colorectal carcinoma HCT116	[39]
Apoptosis via DR5 and caspase 8		Colorectal carcinoma HCT116 Cas9	[40]
Apoptosis via DR5; Inhibition of cell migration, adhesion, and invasion Sensitization to TRAIL-mediated apoptosis via TRAIL-DR5-AMPK signaling	CHOP, ATF4, PERK, eIF2α; AMPK signaling pathway; ROS	Esophageal carcinoma EC109, TE12 C109 xenografts in nude mice	[41]
Sensitization to TRAIL-induced apoptosis via DR5	IRE1α, ATF-6, CHOP	Melanoma Mel-RM, MM200, IgR3, Mel-CV, Me4405, Sk-Mel-28. Mel-FH	[43]
Apoptosis via JNK and ERS (JNK/MAPK/ERK signaling); Inhibition of proliferation, migration and invasion	genes (JNK, ATF6, PERK, LC3B, Bcl-2) proteins (JNK, MAPK, ERK)	Adrenocortical carcinoma SW-13, NCI-H295R adrenocortical carcinoma xenograft in male BALB/c nude mice	[44]
Apoptosis	SEC24A gene	Near-haploid HAP1	[45]
Apoptosis	XBP1s, ATF4, CHOP, LC3B	Prostate cancer PC3, LNCaP Breast cancer MCF-7	[46]

**Table 2:** Phase II clinical trials of mipsagargin.

Cancer Type	Status	Gov Identifier
Glioblastoma multiforme	Completed	NCT02067156
Prostate cancer	Withdrawn	NCT01734681
Advanced Adult Hepatocellular Carcinoma	Completed	NCT01777594
Glioblastoma	Withdrawn prior to enrolment	NCT02876003
Clear Cell Renal Cell Carcinoma	Completed	NCT02607553
Prostatic Neoplasms	Completed	NCT02381236
Hepatocellular Carcinoma	No longer available	NCT02082691

## Abbreviations

AMPK	AMP activated protein kinase
ATF4	activating transcription factor-4
ATF6	transcription factor-6
βAsp-12ADT	8-(βAsp-12-aminododecanoyloxy)thapsigargin
BiP	Binding immunoglobulin protein
CHOP	Transcription factor C/EBP homologous protein
DR5	Death receptor
eIF2α	Eukaryotic Initiation Factor 2
ER	endoplasmic reticulum
ERAD	ER-associated protein degradation
ERK	Extracellular-signal-regulated kinase
FRs	Folate receptors (FRs)
JNK	c-Jun N-terminal kinase 1
JQ-FT	Tg-folate prodrug
LC3B	Microtubule associated protein 1A/1B light chain 3B
Leu-12ADT	8-(Leu-12-aminododecanoyloxy)-8-debutanoyloxythapsigargin
MAPK	Mitogen activated protein kinase
Orai1	Calcium release-activated calcium channel protein 1
PERK	Protein kinase R-like ER kinase
PSA	Prostate specific antigen
PSMA	Prostate specific membrane antigen
SERCA	Sarco/endoplasmic reticulum Ca2+-ATPase
SOCE	Store operated Ca2+ entry
SR	Sarcoplasmic reticulum
STIM1	Stromal interaction molecule 1
T-ALL	T cell acute lymphoblastic leukemia T-ALL
Tg	Thapsigargin
TRAF2	Tumor necrosis factor receptor-associated factor 2
TRAIL	Tumor necrosis factor-related apoptosis-inducing ligand
UPR	Unfold protein response
XBP1	X-box-binding protein 1
XBP1s	Spliced isoform of XBP1
